# Stage-specific miRNAs regulate gene expression associated with growth, development and parasite-host interaction during the intra-mammalian migration of the zoonotic helminth parasite *Fasciola hepatica*

**DOI:** 10.1186/s12864-022-08644-z

**Published:** 2022-06-04

**Authors:** Alison Ricafrente, Krystyna Cwiklinski, Hieu Nguyen, John P. Dalton, Nham Tran, Sheila Donnelly

**Affiliations:** 1 The School of Life Sciences, University of Technology, Sydney, Australia; 2 Molecular Parasitology Laboratory, Centre for One Health and Ryan Institute, National University of Ireland Galway, Galway, Ireland; 3 School of Biomedical Engineering, Faculty of Engineering and Information Technology, University of Technology Sydney, Ultimo, NSW Australia

**Keywords:** *Fasciola hepatica*, Helminth, microRNAs, Newly excysted juvenile, Cathepsin L3

## Abstract

**Background:**

MiRNAs are small non-coding RNAs that post-transcriptionally regulate gene expression in organisms ranging from viruses to mammals. There is great relevance in understanding how miRNAs regulate genes involved in the growth, development, and maturation of the many parasitic worms (helminths) that together afflict more than 2 billion people.

**Results:**

Here, we describe the miRNAs expressed by each of the predominant intra-mammalian development stages of *Fasciola hepatica*, a foodborne flatworm that infects a wide range of mammals worldwide, most importantly humans and their livestock. A total of 124 miRNAs were profiled, 72 of which had been previously reported and three of which were conserved miRNA sequences described here for the first time. The remaining 49 miRNAs were novel sequences of which, 31 were conserved with *F. gigantica* and the remaining 18 were specific to *F. hepatica.* The newly excysted juveniles express 22 unique miRNAs while the immature liver and mature bile duct stages each express 16 unique miRNAs. We discovered several sequence variant miRNAs (IsomiRs) as well as miRNA clusters that exhibit strict temporal expression paralleling parasite development. Target analysis revealed the close association between miRNA expression and stage-specific changes in the transcriptome; for example, we identified specific miRNAs that target parasite proteases known to be essential for intestinal wall penetration (cathepsin L3). Moreover, we demonstrate that miRNAs fine-tune the expression of genes involved in the metabolic pathways that allow the parasites to move from an aerobic external environment to the anerobic environment of the host.

**Conclusions:**

These results provide novel insight into the regulation of helminth parasite development and identifies new genes and miRNAs for therapeutic development to limit the virulence and pathogenesis caused by *F. hepatica*.

**Supplementary Information:**

The online version contains supplementary material available at 10.1186/s12864-022-08644-z.

## Background

Micro(mi)RNAs are a conserved class of small non-coding RNAs (18-25 nucleotides) that regulate gene expression post-transcriptionally [1]. Highly specific binding of the miRNA seed region (nucleotides 2-8) onto a target mRNA 3’UTR can induce complete degradation of the mRNA or obstruct ribosomal scanning to prevent translation [2, 3]. This interaction was first described in the free-living nematode *Caenorhabditis elegans* where the let-7 miRNA was found to be temporally expressed and necessary for the developmental progression of the worm [4], a function that was later found to be conserved in higher organisms [5]. However, with the completion of numerous genome sequences and the identification of many miRNAs in uni- and multi-cellular plants and animals that did not exhibit temporal expression concomitant with development it became evident that miRNAs perform more complex biological functions in regulating gene expression [6, 7]. It is now recognised that most mRNAs are regulated by one or more miRNAs [8] and that they play central roles in co-ordinating gene expression during growth, development, differentiation, metabolism, reproduction, and pathogenesis [7, 9–12].

There is increasing interest in the identification and functional characterisation of miRNA in parasitic worms (helminths). Soil-transmitted intestinal nematodes such as hookworms (*Ancylostoma duodenale* and *Necator americanus*), *Trichuris trichiura* and *Ascaris lumbricoides* infect over two billion people worldwide [13] while flatworms like *Schistosoma* spp. cause ~200 million infections per year [14]. Recent studies have shown that miRNAs are essential not only for the development of parasites within their hosts but for regulation of the complex interactions between them [15–17]. The release of miRNAs by parasites, either freely or as part of their extracellular vesicle cargo, is likely to have been important in the co-evolution of the host-parasite relationship, particularly when the parasites exploit their miRNome to modulate host immune responses [18–20]. The detection of altered expression of unique parasite miRNAs in blood or other tissues could be exploited for the development of novel biomarkers and/or diagnostics of disease [21–24], or may possibly predict or explain the emergence of drug resistance [17].

The flatworm *Fasciola hepatica* (liver fluke) is a zoonotic parasite with a remarkable global prevalence due to its unique capacity to infect and mature in a broad range of mammals [25]. Approximately 2.4 – 17 million people are infected with the parasite and 180 million are at risk of infection which recently impelled the World Health Organisation to classify fasciolosis as a food-borne trematode priority disease [26]. The global economic burden imposed by liver fluke onto the agriculture industry and by human and animal healthcare costs is likely to be in the many billion dollars (US) each year [27].

*Fasciola hepatica* is a very tractable model parasite for investigating the regulation of parasite development and parasite-host interaction because it progresses through distinct stages of growth that are associated with defined host tissues. Moreover, updated draft genomes and extensive stage-specific transcriptomics as well as proteomics (somatic, secretions, tegument and extracellular vesicles) have laid a solid foundation on which to attribute a molecular explanation to processes linked to development [28, 29]. Infection with *F. hepatica* occurs following the ingestion of pasture contaminated with the infective metacercariae that rapidly emerge in the small intestine as newly excysted juveniles (NEJs). Within hours, the NEJs alter their metabolic activities, penetrate the gut wall tissue and migrate to the liver [30, 31]. While migrating within the liver parenchyma the parasite matures, develops digestive and reproductive structures, and undergoes a huge growth phase, doubling in size every few weeks [32–34]. The parasites cause extensive liver damage and haemorrhaging before moving into the bile ducts to complete their maturation and commence the production of progeny (eggs) that are carried with the bile juices into the intestine and liberated onto pasture with the faeces. Accompanying these strict developmental changes as the parasite migrates are highly regulated alterations in gene expression with progressively more genes being expressed with higher fold changes as infection and parasite maturation proceeds [35].

Several independent analyses of the *F. hepatica* miRNome performed on RNA derived from NEJs, mature adults, and adult extracellular vesicles have yielded a collection of ~ 77 *F. hepatica* miRNAs [36–40]. To provide a more detailed and composite picture of the role of miRNAs in *F. hepatica* and its interaction with the mammalian host, we have simultaneously sequenced the small RNAs obtained from the three critical stages of development, including the NEJs that cross the intestine, immature flukes that migrate in the liver parenchyma and adult parasites that reside in the bile ducts, and mapped these against the extensive transcriptome data for these same life cycle stages [30, 32, 35]. Thus, we have expanded and clarified the miRNome repertoire, temporally mapped expression to specific life stages and identified their regulatory gene targets that are essential to parasite growth and development.

## Results and Discussion

### Small RNA sequencing across multiple developmental stages of *F. hepatica* reveals a new repertoire of miRNAs

**Fig. 1:**
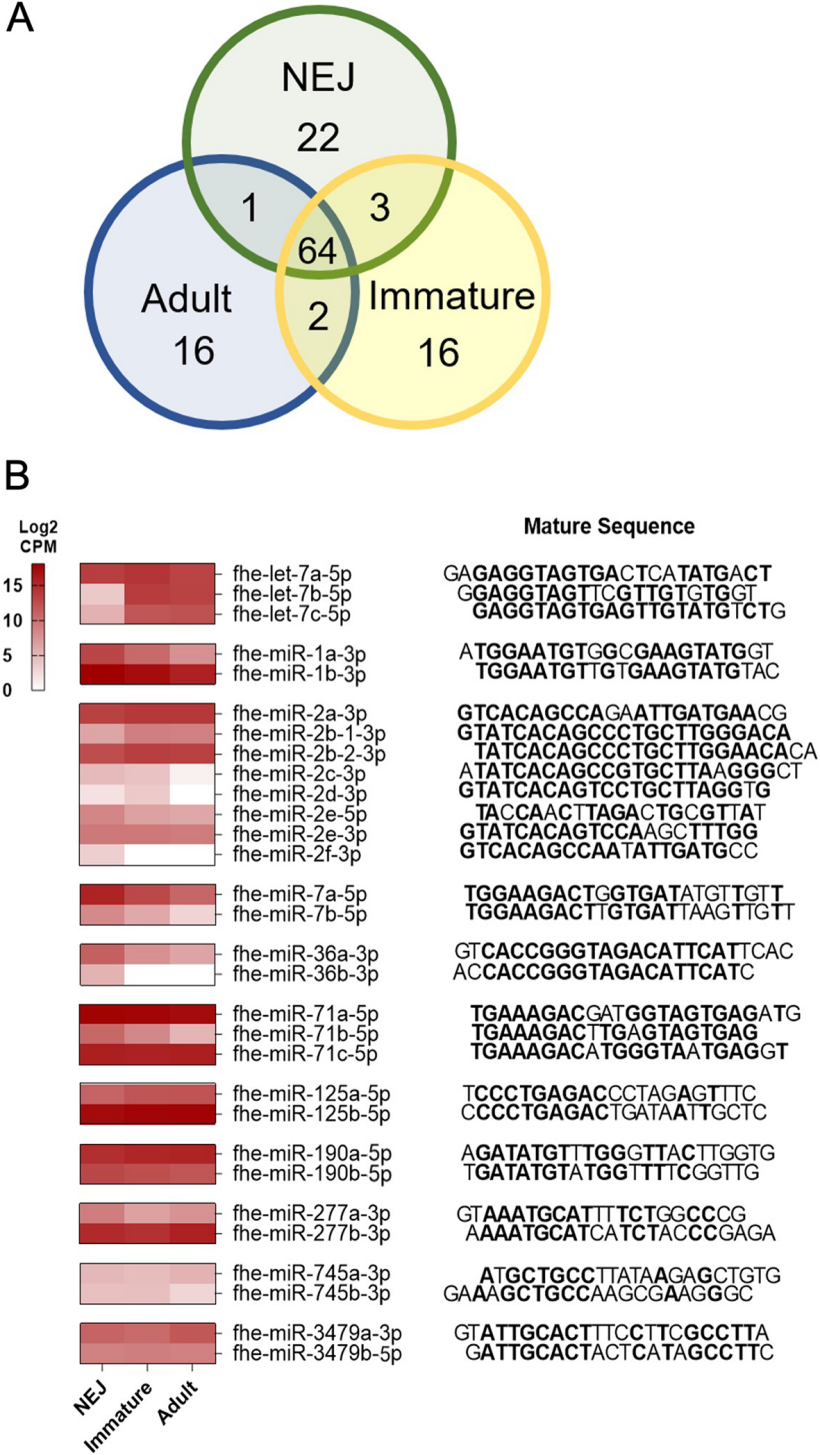
Interrogation of miRBase, published and novel miRNAs establish the *Fasciola hepatica* miRnome. (**a**) Venn diagram of total miRNAs segregated based on presence of the intra-mammalian life stages. (**b**) Normalised reads (CPM) of isomiR families are compared between newly excysted juveniles (NEJ), immature flukes (Juv21d) and adult parasites represented by a heat map. Heatmap depicts low (white) and high (crimson) expression in Log2CPM. Sequences listed 5’ to 3’ for each of the miRNAs and common nucleotides in bold when aligned between one or more miRNAs within a miRNA family.

In depth small RNA sequencing across the three major *F. hepatica* life stages, namely the newly excysted juvenile (NEJ), immature flukes 21-day post infection (Juv 21d) and adult stage parasites identified 124 *F. hepatica* miRNAs (Additional file 1; Table S1). These represent 72 miRNAs that were previously characterised in *F. hepatica* NEJ and adult parasites [41], in addition to 52 newly identified miRNAs, of which three are conserved sequences (fhe-miR-493-5p, fhe-miR-2335-5p and fhe-miR-6613-3p), and 49 were novel miRNA sequences. Of these newly identified sequences, 31 were predicted to be conserved in the *F. gigantica* genome (Additional file; Table S1), although their expression has not been previously reported (Additional file; Table S2) [42]. Based on the revised nomenclature used in this study (Additional file 1: Table S2) and the exclusion of sequences that were not found in all replicates within a single life stage, two of the previously published miRNA sequences were not included in the final compilation of *F. hepatica* miRNAs used in this study. Within this final complement of miRNAs, 22 were unique to NEJ, 16 miRNAs were unique to juveniles and 16 were unique to adults (Fig. 1a).

Following submission of this manuscript, two additional studies were published which report the expanded characterisation of *F. hepatica* miRNAs across multiple life stages (including the NEJ and adult worms) [43, 44]. Combined, these report the discovery of 70 novel miRNA sequences. Of these, two were identical to the *F. hepatica* novel miRNA sequences discovered here (Additional file; Table S2).

In this study and in the two most recently published studies described above, the majority of new miRNA sequences have been identified within the NEJ stage of the parasite life cycle. This is likely due to the different culturing times of NEJs that were used to prepare samples for sequencing. Between the three most recent studies (this manuscript, [43, 44]). RNA was isolated from NEJs excysted in vitro and cultured for 6h, 24h, and 7 days respectively. It has previously been shown that after excystment, the NEJs undergo a period of rapid temporal gene expression with distinct clusters of regulated genes observed at 1h, 3h and 24 h [30]. In addition, the number of genes transcribed at 24h (4,644) greatly exceeds the level of expression at 3h (373). Such vast differences in gene expression would be reflected in corresponding changes to the expression of regulatory miRNAs, explaining the discovery of additional novel miRNAs, as seen here.

The discovery of such a large number of new miRNA sequences is not unprecedented. The identification of a high number of species-specific miRNAs is a trend that has been observed during the assembly of other helminth miRnomes, including *Haemonchus contortus*, which was characterised as having 140 species-specific miRNAs and 44 conserved sequences, and *Schistosoma mansoni*, which expresses 84 species-specific and 28 conserved miRNAs [45, 46].

The simultaneous comparison of miRNA sequences across three life stages revealed the presence of sequence variants of some canonical mature miRNAs (termed IsomiRs), resulting in the expansion of several miRNA families. These variants can be easily dismissed as sequencing artefacts, particularly when identification is dependent on a single sample; accordingly, it is likely that the identification of some *Fasciola* isomiRs were masked in the early discovery projects as only single life stages were analysed. This is particularly exemplified within the let-7 miRNAs where the three initial sequencing studies each described the presence of a single let-7, but each with a different sequence. Examining the expression of let-7 in the different life stages at the same time, we have confirmed the presence of three distinct isomiRs. In addition, the fhe-miR-2 and miR71 families were expanded into eight and three possible miRNAs respectively (Fig. 1B). The remaining nine IsomiR families each contain two miRNAs. Of note, annotation of these isomiRs, specifically the suffix of the miRNA number, was conferred based on the earliest discovery of the miRNA and conservation of the seed region using BLASTN. In our study, the mature sequence of miR-2b was determined with two different precursor structures in different genomic locations and then annotated accordingly.

**Fig. 2:**
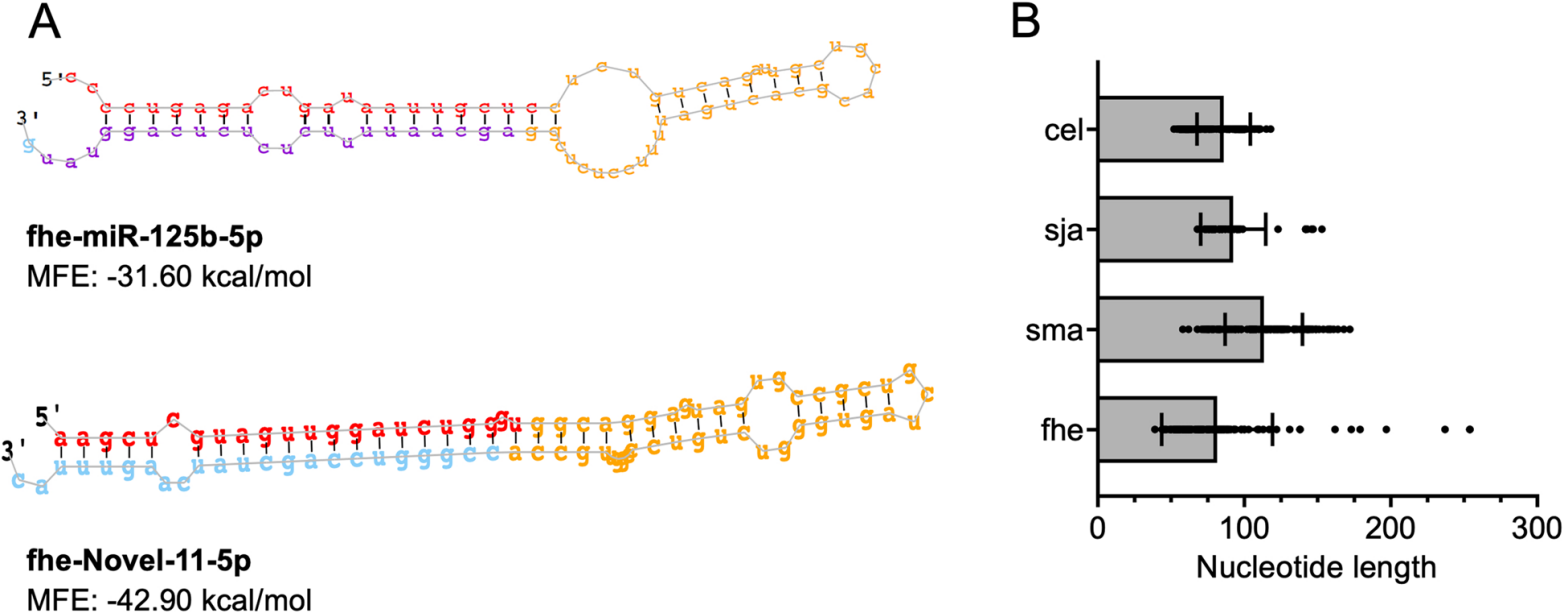
Structural analysis of the *Fasciola hepatica* miRNAs. (**A**) Comparison of precursor miRNA structure of conserved miRNA fhe-miR-125b-5p and novel species-specific miRNA fhe-miR-Novel-11-5p. Nucleotides are colour coded based on predicted mature sequence found in sequenced sample (red), predicted star sequence (blue), predicted star sequence with sequence found in sequenced samples and hairpin loop (yellow) as determined by MiRDeep2. The minimum free energy (MFE) of each miRNA is also displayed. (**B**) Comparison of average nucleotide length of total precursor miRNAs *Caenorhabditis elegans* (cel), *Schistosoma japonicum* (sja)*, S. mansoni* (sma) and *F. hepatica* (fhe)*.*

To validate the authenticity of the novel miRNAs, the precursor sequence structures were compared to published sequences using RNAfold. This analysis revealed conventional precursor miRNA structural features such as a long, truncated hairpin approximately 60 nt long with a 2 nt 3’ overhang, and similar structural stability to known miRNAs, as shown in the calculated minimum free energy (MFE) [47] (Fig. 2a and Additional file 15: Fig. S1). Furthermore, the average nucleotide lengths of the *F. hepatica* precursor miRNAs were similar to those of *S. japonicum, S. mansoni* and *C. elegans* (Fig. 2b). However, we also identified seven *F. hepatica* miRNAs with an uncharacteristically high number of nucleotides outside of the mature sequence, producing precursor structures >150 nt long compared to other worm species. It is interesting that a similar observation has been made for human miRNAs, whereby the evolutionary conserved miRNAs have stable, typical precursor lengths of around 80 nucleotides while the human-specific miRNAs show greater variation in precursor length with some as long as 180 nt [48]. These longer human miRNAs were predicted to regulate a larger number of target genes compared to the conserved miRNAs. Of the seven *F. hepatica* miRNAs with longer precursors, five were specific to trematodes, suggesting that, like human miRNAs, these have evolved for a biological requirement yet to be elucidated.

### Genome location and clusters of *Fasciola hepatica* miRNAs

When mapped to their genome location, most (70%) of the *F. hepatica* miRNAs were found to be intergenic with 86 pre-miRNAs located between protein-coding genes. The remaining miRNAs were intragenic, with 33 pre-miRNAs located within an intron of a specific gene and five embedded within an exon of a specific gene (Additional file 1: Table S1). Having a predominant intergenic miRnome is a shared characteristic with other platyhelminthes, with 92% and 90% of the miRNA complement of *Hymenolepis spp.* [49] and *Schistosoma japonicum* [50], respectively, located in intergenic regions. This contrasts with their mammalian hosts in which >57% of miRNAs are intragenic and transcribed in the same orientation as their host genes [51]. The capacity for the parasites to transcribe their miRNA independently of worm gene expression, suggests an adaptation to efficiently transcribe miRNAs in response to external host signals and to autonomously regulate host genes without significantly disturbing the parasite’s developmental transcriptome.

**Fig. 3:**
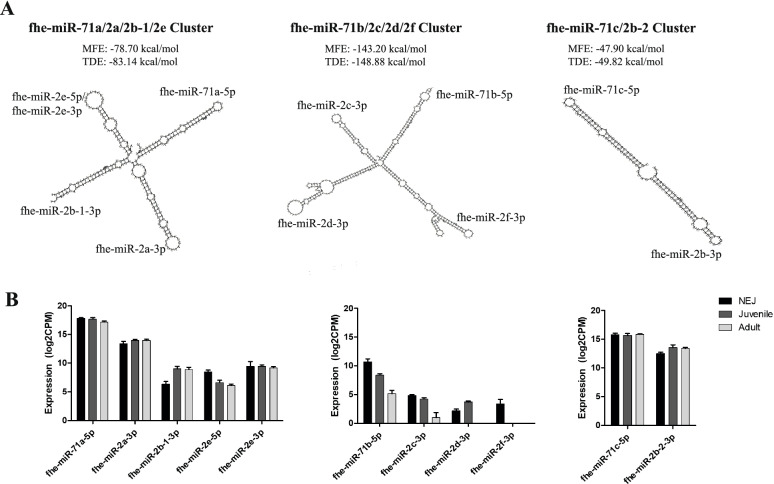
Structure and expression of miRNA clusters of *Fasciola hepatica* miR-71 and miR-2. (**a**) Structural organisation of pre-miRNA sequences using RNAfold to reveal clustering of fhe-miR-71a-5p with fhe-miR-2a-3p, fhe-miR-2b-1-3p and fhe-miR-2e-5p/3p; fhe-miR-71b with fhe-miR-2c-3p, fhe-miR-2d-3p and fhe-miR-2f-3p; and fhe-miR-71c-5p with fhe-miR-2-3p. Each cluster is represented with the minimum free energy (MFE) and predicted thermodynamic ensemble (TDE) of the RNA folding. (**b**) Expression of miR-71 and miR-2 in respective clusters across life stages, Newly excysted juveniles 24h (NEJ), Immature flukes (Juvenile 21dpi) and Adult parasites. Data is presented as normalised reads (CPM) in log2, mean ± SD of triplicate biological samples.

Assessing the genomic arrangement of the 124 miRNAs identified in this study, we found that several of the miRNAs clustered together based on their precursor sequence structure. Notably the miR-71 and miR-2 isomiR families were clustered together, with different sets of the miR-2 isoMiRs clustered with the three miR-71 variants (Fig. 3a). This is consistent with previous observations by Fontenla et al. [38] of a *F. hepatica* miR-71-2 cluster, which is highly conserved across protostomes [52, 53] and has been reported in several platyhelminthes [53–58]. While both miRNAs are derived from the same nascent RNA, investigation of each family member showed that the expression of miR-71 is consistently higher than miR-2 across the life stages (Fig. 3b) implying that miR-71 and miR-2 undergo different rates of processing and functionality. Additionally, the overall expression of the fhe-miR-71a-2a/2b-1/2e and fhe-miR-71c-2b-2 clusters are greater than fhe-miR-71b-2f/2d/2c, suggesting temporal regulation and a putative important role in the transition between the life stages, as indicated for other platyhelminthes [53, 54, 56–59].

### Phylogenetic relationship of *F. hepatica* miRNAs to other parasitic and free-living flatworms.

**Fig. 4:**
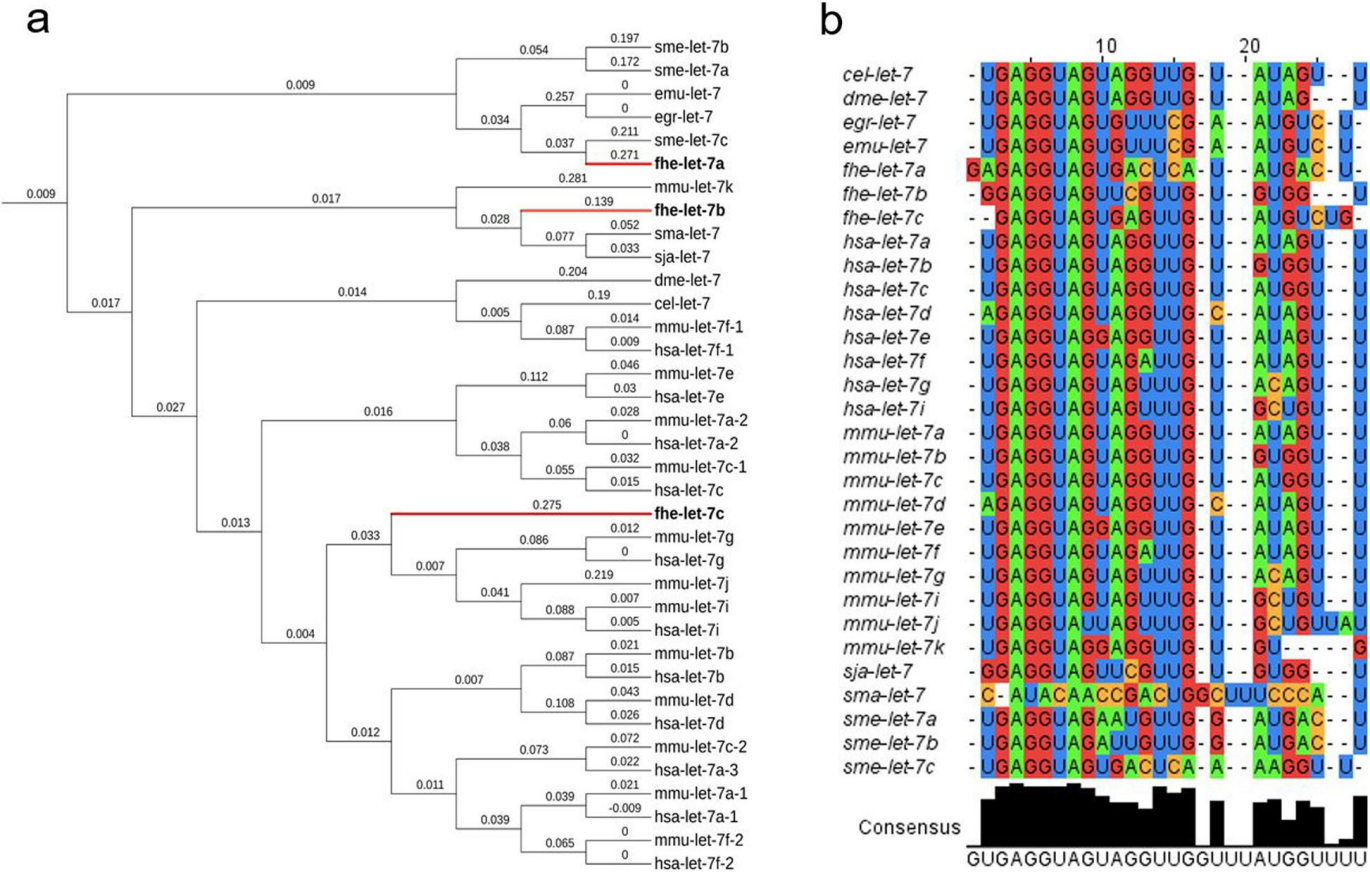
*Fasciola hepatica* let-7 isomiRs are highly conserved as mature sequences yet evolutionarily divergent in comparison to their precursor sequences. (**a**) Phylogenetic tree of let-7 precursor sequences constructed using T-Coffee multiple sequence alignment tool and iTOL v6 (itol.embl.de) with percentage identity scores, 0 = zero sequence difference. (**b**) Sequence alignment of the mature miRNAs of let-7 from *F. hepatica* (fhe) *Caenorhabditis elegans* (cel), *Drosophila melanogaster* – fruit fly (dme), *Echinococcus granulosus* (egr), *E. multilocularis* (emu), *Homo sapiens* – human (hsa), *Mus musculus* – mouse (mmu), *Schistosoma japonicum* (sja), *S.mansoni* (sma) and *Schmidtea mediterranea* (sme) using ClustalW. Homology is illustrated through colour coded nucleotides and consensus nucleotides across all mature miRNA sequences.

Consistent with genome-wide phylogeny of the major parasitic worms [60], phylogenetic analysis based on the precursor miRNA sequences separates the *F. hepatica* miRNome from the sequences derived from *S. japonicum* and *S. mansoni,* which cluster together (Additional file 16: Fig. S2). To explore this divergence more closely, the phylogeny of the highly conserved let-7 miRNA was examined (Fig. 4a). Sequence alignment confirmed a high degree of homology across the let-7 mature miRNAs from human, mouse and fruit fly and the available helminth miRNAs (Fig. 4b). However, in contrast to the total miRNome phylogenetic analysis, the *F. hepatica* let-7 isomiRs are not wholly divergent to other parasites and instead are dispersed throughout the tree; fhe-let-7a shares greater homology with let-7c from the free-living planarian *S. mediterranea* and the parasitic tapeworms *E. granulosus* and *E. multilocularis,* whereas fhe-let-7b is positioned more closely to let-7 from *S. mansoni* and *S. japonicum*. More strikingly, fhe-let-7c has significantly diverged from all worm species and invertebrates, positioned closest to human and mouse let-7g/i/j.

Let-7 is an ancient class of miRNA that has been consistently linked to the temporal regulation of bilaterian developmental biology [5]. Studies into the evolutionary history of let-7 has revealed that the genomic locus of let-7 has been independently restructured between worm species [61]. Let-7 is typically associated to miR-125 as a polycistronic transcript and both miRNAs can act together as key regulators of development [62–64]. However, whereas clustering of let-7 and miR-125 remains conserved in the flatworms *S. mansoni* and *S. mediterranea* [61], these miRNAs appear to be organised separately within different scaffolds of the *F. hepatica* genome, although this may also be due to the fragmentation of the current genome assembly comprised of several scaffolds [38].

Any alteration in the genomic organisation of let-7 between worm species may explain the unique phylogeny of *F. hepatica* let-7 pre-miRNAs. In addition, the marked similarity of *F. hepatica* let-7 to one species and not another suggests diversification of its gene targets and thus biological functions. Previous analysis of precursor sequences of miR-125b-5p provides support for this evolutionary adaptation, as the structure of the *F. hepatica* miR-125b-5p is also more closely related to the human miR125-b, than to the Schistosome miR-125b, and is predicted to regulate expression of the same host genes as the human miRNA [20]. The divergence of the *F. hepatica* miRNA network could contribute to the ability of this parasite to more broadly regulate host genes and consequently infect a wide range of mammalian hosts.

### *Fasciola hepatica* miRNAs are temporally expressed during the parasite’s development from newly excysted juvenile to adult worm

**Fig. 5:**
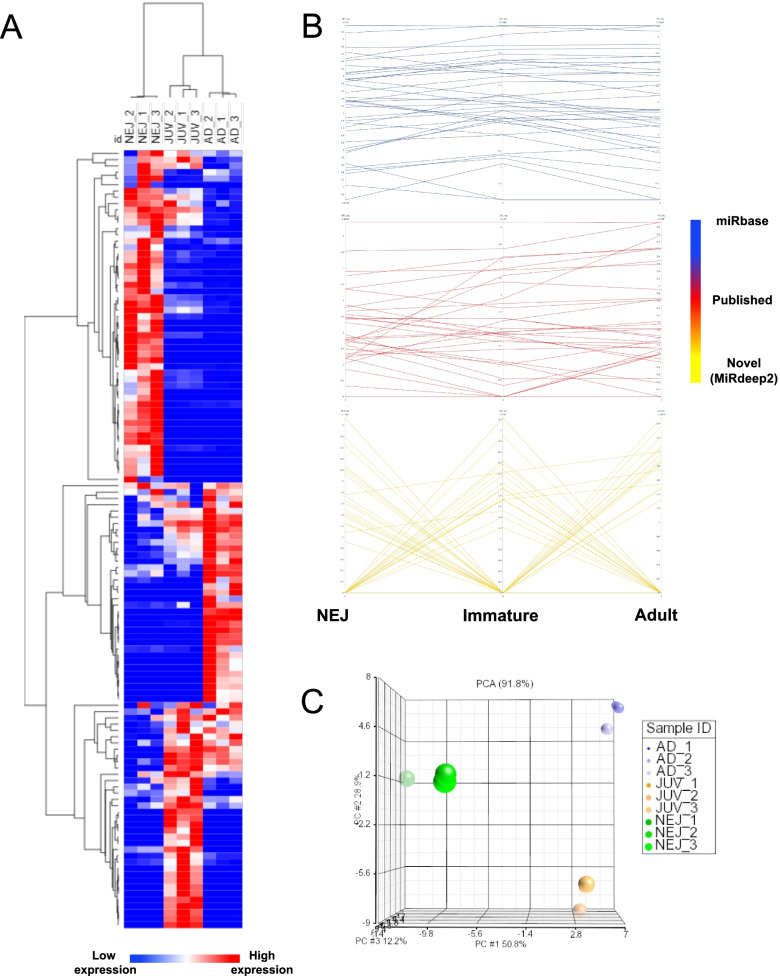
The *Fasciola hepatica* miRnome displays temporal expression during the life cycle within the mammalian host. (**a**) Heatmap of the expression of the miRnome across triplicate RNA samples of newly excysted juveniles (NEJ), immature fluke (JUV) and adult parasites (AD) where minimum expression (low expression – blue) and maximum expression (high expression – red) of the individual miRNAs is represented. Hierarchical clustering of the average values of miRNAs and sample types was performed using One Pearson correlation. (**b**) Parallel coordinates of *F. hepatica* miRNA expression (logCPM) separately analysed based on category; miRBase miRNAs (blue), other published non-miRBase miRNAs (red) and novel miRNAs determined in this study (yellow). (**c**) Principal components analysis (PCA) plots of the total miRNA expression across all sample types and miRNAs in 3D (From left to right – front view, side view, top view). Size of the nodes depicts total miRNA input (miRNA presence) between sample types NEJ (green scale), immature flukes (orange scale) and adult parasites (blue scale).

Hierarchical clustering reveals that the transcriptional profile of the *F. hepatica* miRNAs mirrors that of the gene transcriptional profile, both of which display differential expression as the parasite develops and matures from the invasive NEJ to the sexually mature adult worms (Fig. 5a; Additional file 3: Table S3; [35]). In particular, the NEJ miRNAs exhibit a profile of miRNA expression that is quite distinct from the other stages, with the immature and adult parasites displaying a more similar profile and abundance of miRNA expression (Fig. 5 a, c). This pattern of expression is consistent with the major developmental and growth processes that the parasite is undergoing following invasion and subsequent migration through the mammalian host [30, 32, 35]. The similarity between the miRNA and gene transcriptional profiles aligns with the expectation that miRNAs regulate the gene networks involved in developmental progression.

Further interrogation of *F. hepatica* miRNA transcription by parallel co-ordinate plot analysis revealed an unexpected distinction between sets of miRNAs (Fig. 5b). The miRNAs that had been previously characterised showed very little fluctuation in their transcriptional profile across the three life stages. In contrast, the novel miRNAs described here displayed differential expression between the life stages. The marked difference between the expression profiles of these miRNAs can be partly explained by the nature of miRNA discovery tools that rely on species homology to predict precursor structures within the genome. As the miRNAs derived from miRBase and other published sources were some of the earliest realisations of the *F. hepatica* miRnome, their identification was largely informed by highly conserved miRNAs that are likely necessary for critical physiological functions and thus required throughout the growth and development of the parasite. Since these initial studies were performed, two assemblies of the *F. hepatica* genome have become available, both of which have been revised with technological advances, and the number and quality of worm miRNA sequences used as reference input has vastly expanded supporting the identification of novel miRNAs in this study.

### Identification of miRNA gene targets, correlated to the parasite’s transcriptome, reveals the global regulation of stage-specific developmental processes.

To elucidate the functional role for the parasite miRNAs in regulating the developmental processes, gene targets for each miRNA were predicted from the 3’UTR regions of genes within the *F. hepatica* genome using miRanda and TargetScan (Additional file 4: Table S4; Additional file 5: Table S5). Genes that were commonly selected by both tools produced a total of 8,386 predicted gene targets (Additional file 6: Table S6). The frequency distribution of the number of targets per miRNA showed that the majority of the *F. hepatica* miRNAs were predicted to have 20-100 targets, with an average of 67 gene targets per miRNA (Additional file 17: Fig. S3). Five miRNAs, of which four (fhe-miR-1a-5p, fhe-miR-71a-5p, fhe-pubNovel-2-5p and fhe-Novel-15-3p) were most abundant in the NEJ life stage, were predicted to have >200 targets.

**Fig. 6:**
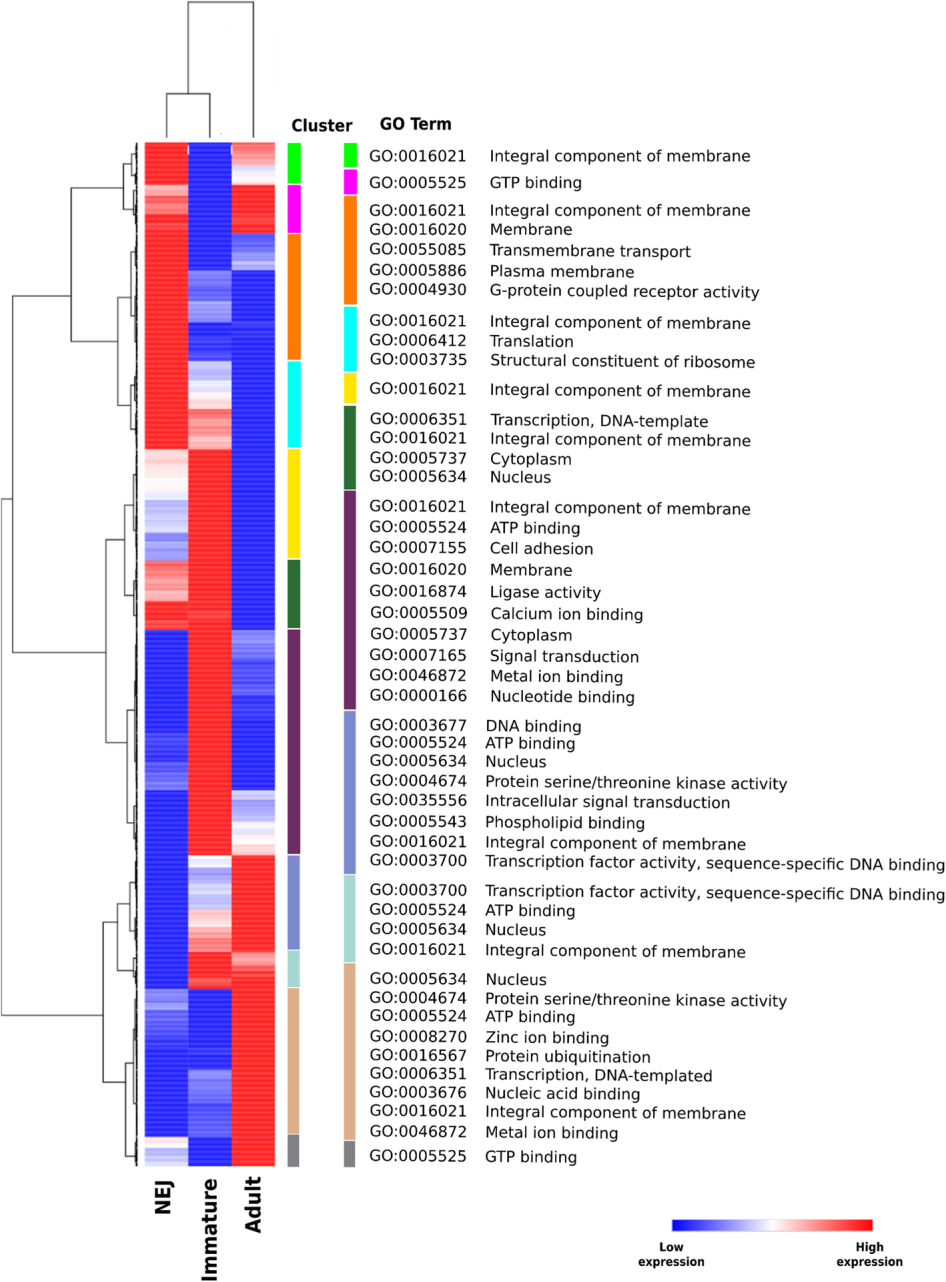
Hierarchical clustering of targets shows dynamic miRNA-induced regulation of biological processes across *F. hepatica* life stages. Heatmap of gene expression (TPM) of targets that are negatively correlated to the transcription of associated miRNAs. Hierarchical clustering of gene target expression performed using One Pearson correlation on the average TPM of each mRNA cross the sample types. Gene expression presented from a scale of the minimum expression (low expression - blue) versus the maximum expression (high expression - red) of the specific gene across the sample types. Each cluster was analysed for gene ontology (GO) enrichment using hypergeometric tests, and GO terms selected on adjusted *P* value <0.001. Order of GO terms for each cluster organised from most significant to least significant.

Of the predicted targets, 2,427 genes displayed transcriptional levels that negatively correlated to the expression of their corresponding regulatory miRNA (Additional file 18: Fig. S4 and Additional file 7: Table S7). Hierarchical clustering of these genes based on their expression within the three life stages revealed possible miRNA-specific regulation of key processes (Fig. 6; Additional file 8: Table S8). While the expression of genes associated to the integral component of the membrane was present in clusters associated to each of the life stages, it appears to be the predominant biological process in the NEJ parasites (clusters 1-4). As the parasites develop from NEJ to immature fluke, there is evidence of heightened expression of genes associated with nucleotide synthesis, transcription and translation, energy regulation, cell signalling and proteolysis (clusters 5-7). The increase in the expression of these genes reflects a significant developmental transition for the parasite as it acquires the ability to feed on host tissue and blood, migrate and undergo rapid periods of growth. These same biological processes are also evident in the adult parasites as they continue to feed and grow (clusters 8-11). However, specific to the adult stage is the presence of zinc ion binding, which is consistent with the employment of metallo-peptidases in the process of egg development [59]. This general alignment with the developmental stages of the parasite, supports the evidence from *C. elegans* and other nematodes, and shows that *F. hepatica* miRNAs are differentially expressed to stage-specifically regulate the transcriptome to ensure the biological processes required for each developmental stage are switched on and off as necessary.

**Fig. 7:**
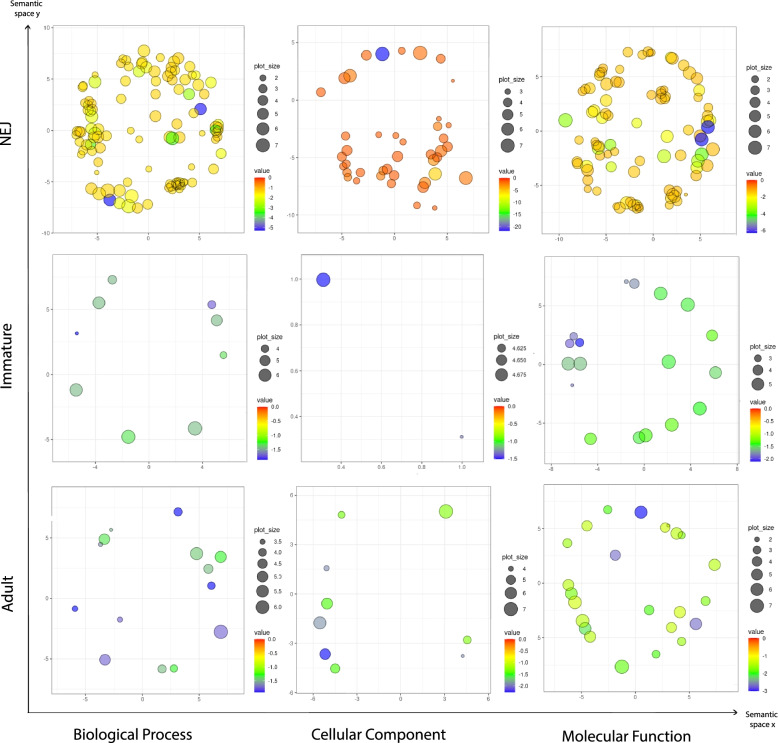
The NEJ specific miRNAs regulate a significantly higher number of targets compared to the immature or adult specific miRNAs. Semantic similarity charts on gene ontology (GO) enrichment for biological processes, cellular components and molecular functions of genes regulated by miRNAs specifically expressed in each of the intra-mammalian life stages; newly excysted juveniles (NEJ), immature fluke and adult parasites. Enrichment analysis performed using hypergeometric tests and GO terms with <0.05 adjusted *P* value selected. Charts constructed using SimRel for semantic similarity measure, where nodes are colour coded based on *P* value and node size representing GO presence.

Although the number of miRNAs that were identified as exclusive for each life stage was very similar (22 NEJ; 16 Immature fluke; 16 Adult), there was a stark contrast in the number and variety of biological activities being targeted in NEJs when compared to the immature and adult parasites (Fig. 7; Additional file 9: Table S9). This outcome reflects the total number of gene targets associated to each of the miRNAs. Surprisingly, a total of 559 unique targets were identified for the 22 NEJ miRNAs, while the immature and adult parasite specific miRNAs were found to have 22 and 56 unique gene targets, respectively (Additional file 10: Table S10). It has been suggested that when the expression of multiple genes is simultaneously inhibited by a single miRNA, as in the NEJs, this miRNA is likely serving to enforce a cell or organ identity [65]. This was elegantly demonstrated in zebrafish, where the expression of miR-430 at the onset of zygotic transcription results in the co-ordinated clearance of hundreds of maternal genes thus regulating morphogenesis and controlling temporal identity [66]. Similarly, the NEJ specific miRNAs may suppress the expression of a broad numbers of genes with a range of biological functions until the cues from the internal environment of the host signals a requirement for these genes to support a developmental transition. An examination of the broad pattern of gene expression through the life stages would support this proposition, as there is a much higher number of genes that are similarly increased in expression in the immature and adult worms compared to the NEJs [35].

### MiRNAs regulate parasite metabolic pathways

Our previous analysis of the *F. hepatica* stage-specific transcriptomes has demonstrated that tight regulation of metabolic pathways is critical to support the physiological changes to the parasite as it matures and adapts to changing microenvironments during its migration through the host [30, 32, 35]. Interrogation of the negatively correlated gene targets of the *F. hepatica* miRNome revealed that 64 miRNAs putatively target genes associated with a variety of metabolic processes (Additional file 11: Table S11). While 58 of the miRNAs were predicted to have a broad effect across multiple pathways, six miRNAs were exclusively associated with specific metabolic pathways, namely (**a**) Energy metabolism: fhe-miR-307-5p, (**b**) Amino acid metabolism: fhe-Novel-11-5p, (**c**) Transcription: fhe-Novel-28-5p, (**d**) Translation: fhe-miR-125b-5p & fhe-Novel-26-5p, (**e**) Signal transduction: fhe-miR-9389-5p.

**Fig. 8:**
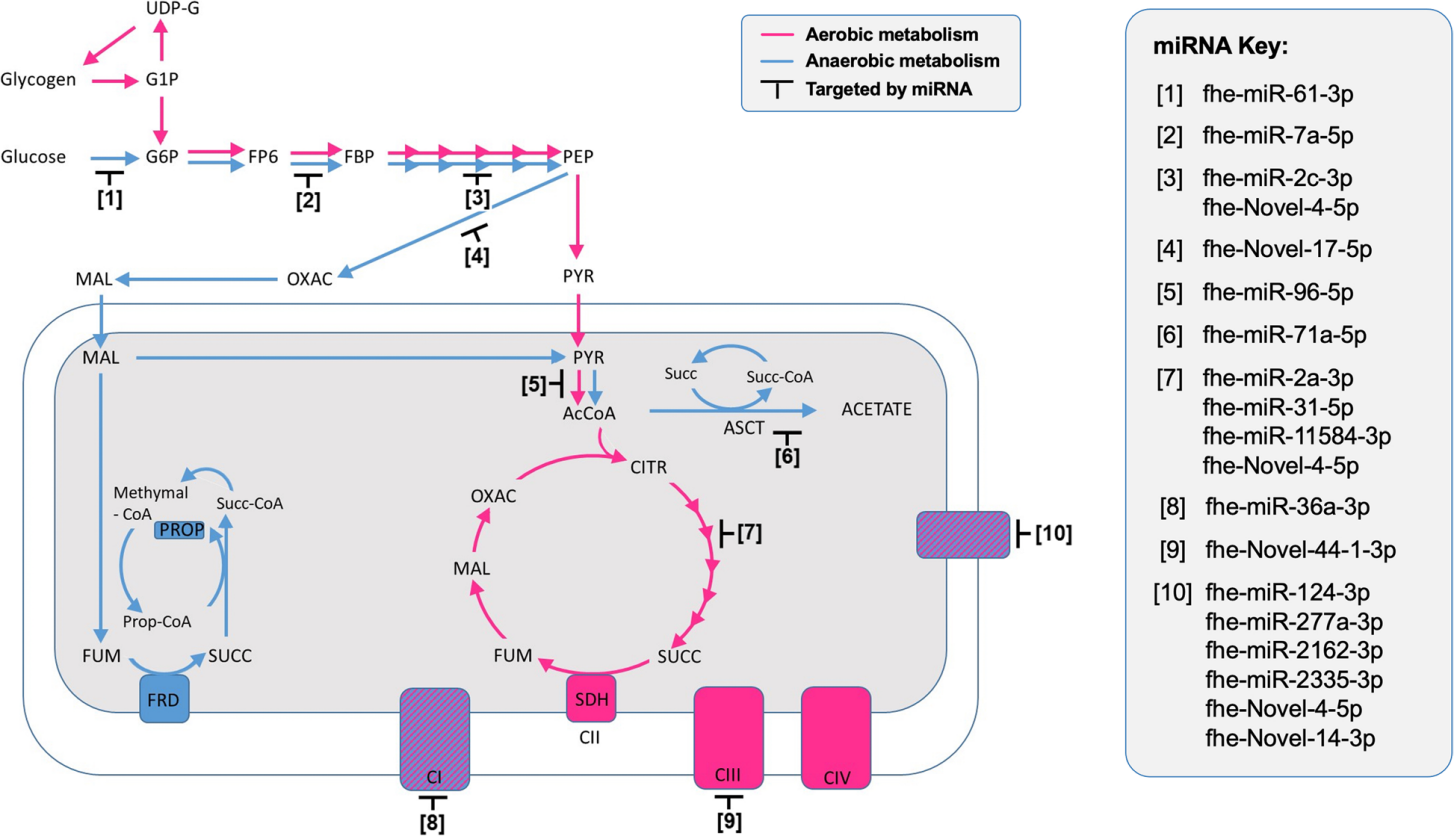
miRNAs target genes involved in aerobic metabolism. Graphical representation of the pathways involved in the glycolysis/gluconeogenesis, TCA/Krebs cycle and oxidative phosphorylation KEGG pathways modified from the figure by Tielens and van Hellemond (2021). The black bars and numbers highlight position in the pathways that the miRNAs are targeting, based on the KEGG analysis carried by Cwiklinski et al. (2015, 2018, 2021). Abbreviations: AcCoA: acetyl-CoA; ASCT: Acetate:succinate CoA transferase; CITR: citrate; FP6: fructose 6-phosphate; FBP: fructose 1,6-bisphosphate; FRD: fumarate reductase; FUM: fumarate; G1P: glucose 1-phosphate; GP6: glucose 6-phosphate; MAL: malate; Methymal-CoA: methylmalonyl-CoA; OXAC: oxaloacetate; PEP: phosphoenolpyruvate; PROP: propionate; Prop-CoA: propionyl-CoA; PYR: pyruvate; SDH: succinate dehydrogenase; SUCC: succinate; Succ-CoA: succinyl-CoA; UDP-G: uridine biphosphate glucose.

The accessibility of glycogen/glucose and oxygen are the primary parameters that drives *F. hepatica* to adjust its energy metabolism. Following excystment, the NEJs must begin the transition from catabolism of glycogen stores to the synthesis of glucose/glycolysis from host macromolecules. As the parasite grows and moves deeper into host tissue, oxygen diffusion within the parasite becomes limited resulting in a gradual switch from aerobic to anaerobic metabolism. The key pathways involved in this developmental process are the glycolysis/gluconeogenesis, TCA/Krebs cycle and oxidative phosphorylation pathways [67]. Our analysis revealed that 17 miRNAs targeted keys genes within these critical energy metabolic pathways, including six isomiRs and members of the mir-71-2 cluster described above (fhe-miR-2a-3p, fhe-miR-2c-3p, fhe-miR-7a-5p, fhe-miR-36a-3p, fhe-miR-71a-5p, fhe-miR-277a-3p) (Fig. 8 and Additional file 12: Table S12). However, there was no evidence of a clear stage specific switch in the expression of any of the miRNAs or their targets. This suggests the miRNAs play a role in fine-tuning the metabolic pathways throughout the development of the parasite in the mammalian host.

### Seven miRNAs are abundantly expressed by all three *F. hepatica* life stages

**Fig. 9:**
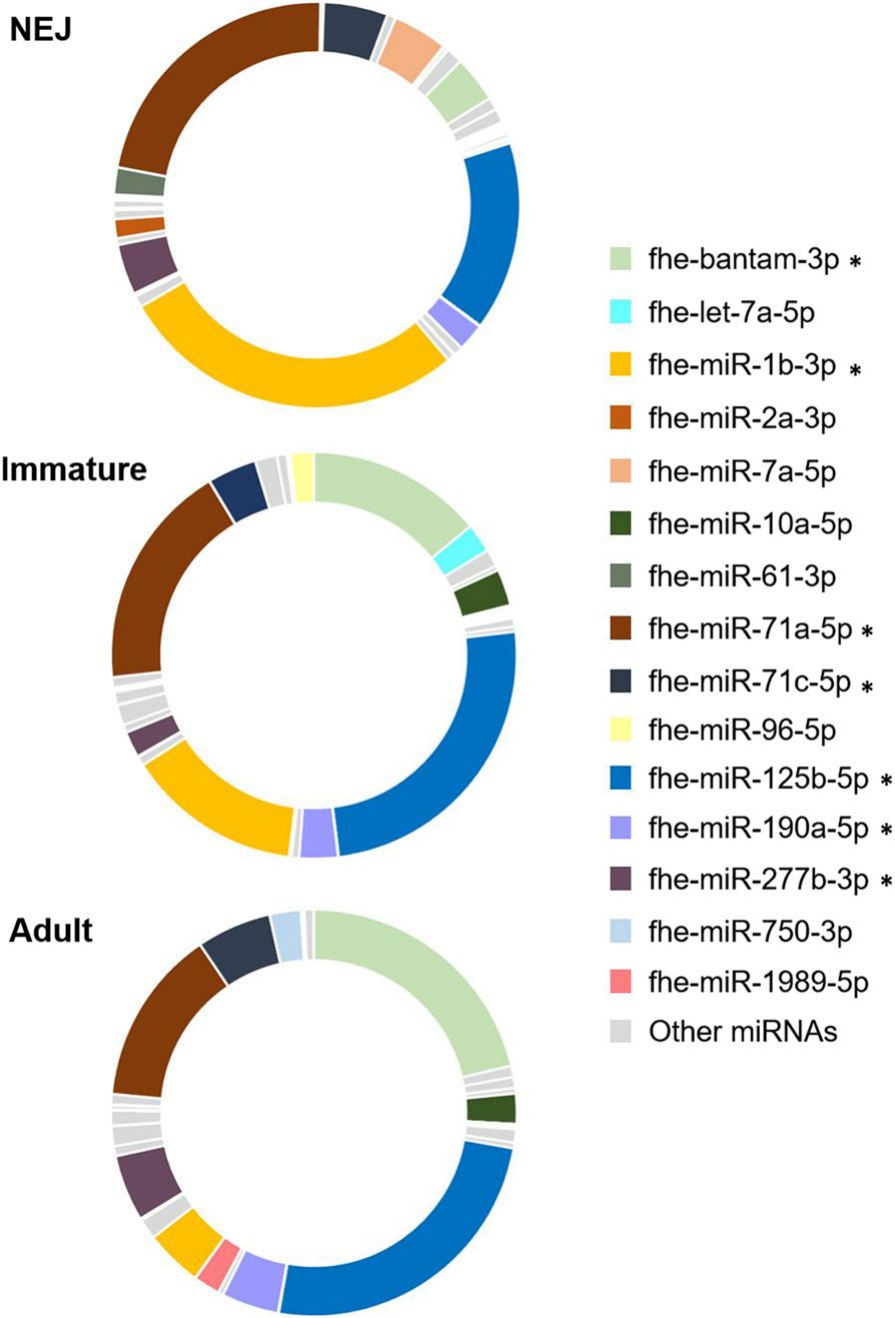
Top 10 abundant miRNAs across intra-mammalian life stages share similar enrichment. Pie charts of the percentage of individual miRNA expression (CPM) across newly excysted juveniles (NEJ), immature flukes and adult parasites. Only the top 10 most abundant miRNAs for each life stage are colour coded. MicroRNAs that are present in the top 10 most abundant in all three of the life stages are denoted with an asterisk *.

While the overall miRNA profiles were unique for each developmental stage examined, seven miRNAs were amongst the ten most abundant miRNAs in every life stage: bantam-3p, miR-1b-3p, miR-71a-5p, miR-71c-5p, miR-125b-5p, miR-190a-5p and miR-277-3p (Fig. 9). This is consistent with earlier studies of the *F. hepatica* miRNAs, which identified the same miRNAs in NEJs [38], adult parasites and within the extracellular vesicles secreted by adult parasites [39, 40]. Similarly, bantam-3p, miR-71-5p, miR-2a-3p were found to be enriched within eight life stages of *F. gigantica* (egg, miracidia, rediae, cercariae, metacercariae, juvenile and adult stages). The high degree of expression of a small number of miRNAs is not specific to *Fasciola* spp. and has been reported in a range of other platyhelminth miRNA studies [40, 68–70].

Although these seven are the most highly enriched across all life stages, their relative abundance changes as the parasite develops, grows, and matures (Fig. 9). Examining the gene targets for these miRNAs (Additional file 13: Table S13) provides some insight into their requirement at different stages. The biological implications are most apparent for bantam-3p, as only one gene target, cathepsin L3 (FhCL3), was identified for this miRNA. Our previous transcriptome analysis revealed that FhCL3 is abundantly secreted by NEJs to facilitate the migration of the parasite through the intestinal wall and the liver [30, 35]. As the parasite matures and moves from tissue-feeding to blood-feeding, the expression of FhCL3 is switched off and other family members, FhCL1 and FhCL2, with altered substrate specificity becomes the predominant peptidase. Thus, the increased abundance of bantam-3p during the immature and adult stages could ensure that the expression of FhCL3 remains downregulated. Of the other 34 cathepsin peptidases identified within the *F. hepatica* genome [71], only one other sequence (Cathepsin B-like protease) was identified as a gene target for the parasite miRNA fhe-Novel-14-3p which is specifically expressed in the NEJ stage.

The function of the genes targeted by miR-125b and miR-190a-5p are less clear and no gene targets negatively correlated to the life stage transcriptome were predicted for miR-1b-3p, which was most abundant in the NEJs. The developmental contribution of these differentially expressed miRNAs will only become evident with more understanding of the biochemistry and activity of the parasite proteins encoded by the genome, although these miRNAs could also play a role in host-parasite interactions and, therefore, have gene targets within host cells. We have previously reported the presence of *F. hepatica* miR-125b within the peritoneal macrophages of mice infected with the parasite 24 hours earlier. Furthermore, the discovery that this miRNA was loaded onto the mammalian Ago2 protein within host macrophages [20, 72], and subsequently regulated the expression of inflammatory cytokines, was hypothesised as a stage specific strategy by which the NEJs disarm the host inflammatory response to protect the NEJs as they migrate from the intestine to the liver and establish infection [20].

### A select number of genes with stage specific activities are highly targeted by multiple miRNAs.

**Fig. 10:**
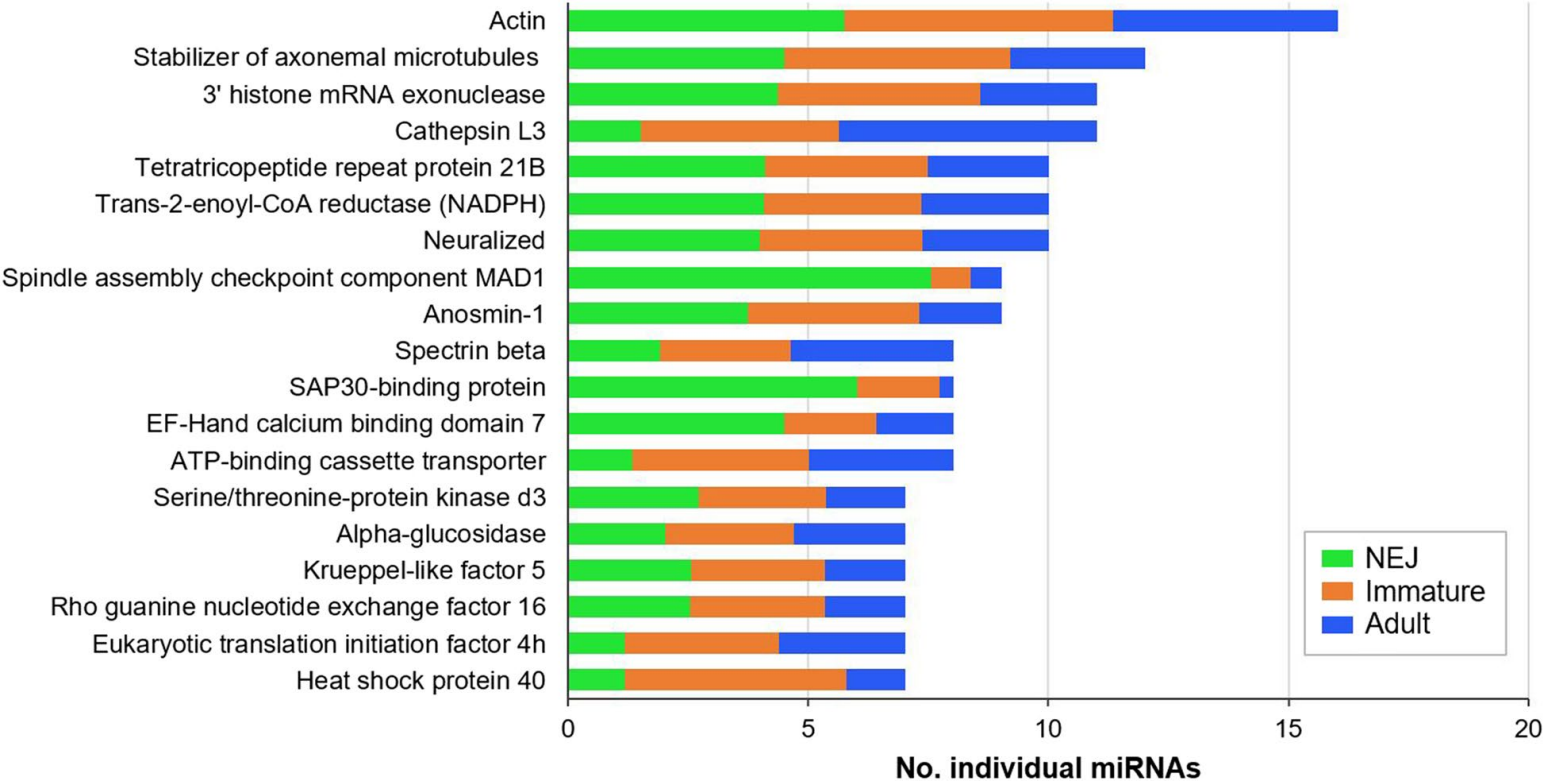
Genes associated to parasitism, development and RNA and DNA regulation are frequently targeted by *F. hepatica* miRNAs. Specific genes with ≥7 different individual miRNA interactions are organised by frequency of interaction from highest to lowest number of individual miRNAs. Columns are colour coded based on percentage of the total miRNA expression of the individual miRNAs according to life stages, newly excysted juveniles (NEJ) (green), immature fluke (orange) and adult parasites (blue), to represent level of miRNA-mediated regulation of the gene within each life stage.

Despite the high level of specificity shared between miRNAs and mRNA interactions, a significant degree of redundancy in miRNA can occur in which several miRNAs target a single gene. The combined action of multiple miRNAs ensures more effective regulation of gene expression [73, 74]. While most gene targets within the parasite transcriptome were negatively regulated by up to 6 miRNAs each, 19 genes are putative targets of more than seven miRNAs (Fig. 10; Additional file 7: Table S7). The scope of regulation for these genes infers a critical importance to the timing of their expression.

Some of these genes (Actin, Stabiliser of axonemal microtubules, Anosmin, Exonuclease) are involved in normal growth and development processes and are therefore regulated consistently by similar numbers of miRNAs throughout all intra-mammalian life stages. In contrast, FhCL3 is predominantly targeted by miRNAs within the immature and adult parasites suggesting this protein is specifically required by the NEJs (Fig. 10). Together with the observation that one of the most abundant miRNAs in immature and adult flukes (bantam-3p; Fig. 9) also solely targets this gene, further suggests that regulation of this cysteine peptidase is critical to the transition from NEJ to immature worm. A similar profile of regulation was found for ATP-binding cassette transporter, heat shock protein (HSP) 40, and Eukaryotic translation initiation factor 4h, with all three genes primarily targeted by multiple immature and adult miRNAs. Although the biological functions for these proteins in the life cycle of *F. hepatica* have not yet been elucidated, evidence from other organisms suggest they provide an increased resilience against numerous environmental stressors [75–77], which is critical for the NEJs as they excyst in the harsh environment of the gut, migrate through the intestinal epithelium and divert host signals of tissue damage and inflammation as they travel to the liver tissue.

In contrast, the expression of SAP30-binding protein, is primarily regulated by multiple miRNAs during in NEJs, a level of regulation which is significantly reduced during the immature stage and almost absent in the adult worms. This protein is a component of the histone deacetylase (HDAC) which has been shown to have fundamental roles in maintaining the viability of *S. mansoni* adult worms and egg production [78]. Therefore, the scarcity of SAP30 targeting by miRNAs in juveniles and adults suggests a critical need in the biological process of egg production, relevant only to the mature stages of the worm.

The only other gene which was similarly highly regulated during the NEJ stage was spindle assembly checkpoint component (SAC) MAD1. It has been shown that SACMAD1 is completely absent in planarian *S. mediterranea*, which is hypothesised to reflect the evolutionary development of core cellular mechanisms, and thus linked to the regenerative ability of these primitive worms [79]. The biological role for SAC components has not been explored in *F. hepatica* but we speculate that the significant miRNA-mediated suppression of SACMAD1 in NEJs relates to the enhanced proliferation of neoblasts observed during the 24h after excystment [30], as these are the same cells that mediate planarian regeneration.

## Conclusions

The *F. hepatica* genome is one of the largest pathogen genomes sequenced to date. With no evidence of genome duplication or increased repeat regions, it has been proposed that much of the non-coding portion of the genome is involved in gene regulation, reflecting a need to tightly control the complex life cycle and variety of developmental stages for this parasite [35]. MicroRNAs have been well characterised as one form of non-coding RNA that is critical in shaping worm development in response to a variety of host and environmental conditions, through the regulation of co-ordinated expression of mRNA transcripts. Although, we do not have a fundamental understanding of their regulatory mechanisms, our study has shed new light on the possible functional roles for many of these parasite miRNAs through the identification of target genes within the parasite transcriptome that are critical in the transition from NEJ to adult.

However, for several of the *F. hepatica* specific miRNAs, the number of predicted genes were reduced to zero after correlation with the parasite transcriptome. Although these miRNAs could be specifically utilised in the parasite’s interaction with host cells, it is also possible that this outcome reflects the limitations of the currently available target prediction tools (which are based on mammalian seed interactions). When applied to non-model organisms (such as helminths) current assumptions for target prediction using the seed region may not account for inherent differences in the biochemistry of species-specific miRNA and mRNA interactions. In some cases, there is a requirement for binding “beyond the seed region” to initiate silencing. At this stage we cannot discount an alternative mechanism of gene target regulation for these specific miRNAs. Despite these shortcomings, the use of an established bioinformatics approach allowed the identification of many gene targets within the parasite transcriptome, and these were mapped to significant biological pathways. However, a sizeable proportion of *F. hepatica* genes remain uncharacterised.

Despite these limitations, this study provides an expanded compendium of miRNAs that are utilised by the parasite as it matures within the mammalian host. The temporal expression of these miRNAs across three life stages and the identification of corresponding gene targets alluded to a role in the regulation of critical developmental processes and metabolic pathways. Our findings set the foundation for future targeted strategies to fully determine the critical nature of the contribution that miRNAs and corresponding gene targets make towards the maturation of the parasite and thus the successful infection of mammalian hosts.

## Methods

### Sample preparation of parasite material

*Fasciola hepatica* metacercariae (Italian isolate), sourced from Ridgeway Research Ltd (UK) were used for excystment and 24h culture of newly excysted juveniles (NEJ) [30], and oral infections of mice and sheep, to recover 21-day immature flukes [32] and adult parasites [80], respectively, as previously described. Total RNA was extracted from the three *F. hepatica* life stages in triplicate using the miRNeasy mini kit (Qiagen) according to the manufacturer’s instructions, in a final elution of 50µl RNase-free water (Number of parasites per replicate for each stage: NEJ: 1000; Immature fluke: 19; Adults: one). RNA integrity and concentration were verified using the 260/280 LVis plate functionality of the PolarStar Omega Spectrophotometer (BMG LabTech) and the Quant-iT RiboGreen RNA Assay Kit (ThermoFisher Scientific).

### Sequencing

Library preparation of total RNA from samples was performed by ArrayStar using the Small RNA library Prep Set for Illumina and sequenced using Illumina NextSeq 500. From FASTq sequencing files, adaptor sequences were excised and filtered for low quality (<20 phred score) sequences and low length sequences (<18 nt) using bioinformatic tool CutAdapt (v3.4).

### Bioinformatics – Interrogation, Quantification and Annotation of *F. hepatica* miRNAs

Mature miRNA sequences from the miRBase *F. hepatica* repository (Fhepatica_v1, miRBase v21) and other published sources as described in Ricafrente et al. [41] were aligned to cleaned reads using Bowtie (v1). Interrogation and quantification of novel *F. hepatica* miRNAs was performed using miRDeep2 (v2). Precursor miRNA structures were predicted using the *F. hepatica* genome generated by WormBase Accession (PRJEB25283) and compared to mature miRNAs sequences from *F. hepatica* (miRbase and published literature), alongside mature miRNAs from trematodes *Schistosoma japonicum* (ASM15177v1), *Schistosoma mansoni* (WTSI5), and non-parasitic nematode *C. elegans* (WBcel235). Mature miRNA sequences that were highly similar were considered to be isomiRs when the expression of sequence variants (that were not already considered isomiRs in previous studies) could be differentiated between the life stages by at least a 2-fold change in expression. The bioinformatic workflow is shown in Additional file 19: Fig. S5. Sequences were only considered to be novel when read counts were present in all three triplicates of at least one of the life stages and when the genome coordinates of its predicted precursor miRNA structure were the same within the triplicate for that life stage.

To address the different annotation styles that were historically used to describe the *F. hepatica* miRnome, miRNAs in this study were annotated using methodology featured in [81] to minimise duplication of miRNA identities. As a result, some previously identified miRNAs were renamed based on the best fitting homology to conserved seed and precursor structures using BLASTN (Additional file 2: Table S2). IsomiRs were annotated with a suffix that matched conserved miRNAs in miRBase using BLASTN or based on chronological discovery from earliest to most recent identification in other published works. Novel miRNAs were annotated based on conserved miRNAs in miRBase using BLASTN. All other novel miRNAs that were not found to be conserved and are species specific were annotated as follows (using miRBase convention); miRNAs were named fhe-Novel-x or if homologous to a previously published non-conserved miRNA then named fhe-pubNovel-x, and arbitrarily numbered in sequence. 5p or 3p was added to differentiate between miRNAs that originate from the 5’ or 3’ halve of the precursor hairpin. Finally, miR-X-1-5p and miR-X-2-5p was used to denote two miRNAs with the same miR-X mature sequence, but with 2 different genome locations

### Phylogenetic Analysis

To determine phylogeny of the trematode miRNAs, a phylogenetic tree of all precursor miRNAs of *F. hepatica* from this study, and precursor sequences of *S. japonicum* (ASM15177v1) and *S. mansoni* (WTSI5) from miRBase (v22) were aligned using the multiple sequence alignment program T-coffee (www.ebi.ac.uk/Tools/msa/tcoffee) using default parameters for the neighbour joining method and the phylogenetic tree visualised using iTOL (v5) [82]. Using similar methods, the phylogeny of Let-7 precursor miRNAs was determined using precursor sequences from miRBase repository (v22); human – *Homo sapiens* (GRCh38), mouse – *Mus musculus* (GRCm38), fruit fly - *Drosophila melanogaster* (Release_6), alongside worm species *C. elegans* (WBcel235), *Echinococcus granulosus* (ASM52419v1), *Echinococcus multilocularis* (WTSI3) *S. japonicum* (ASM15177v1), *S. mansoni* (WTSI5), *Schmidtea mediterranea* (WUSTL3.1), compared to *F. hepatica* let-7 isomiRs from this study. Conservation of the mature miRNAs of the respective precursor miRNAs were compared using T-coffee, and ClustaIW sequences generated using Jalview. To determine homology within *Fasciola* spp, precursor sequences of novel miRNAs determined in miRdeep2 analysis were compared to the *F. gigantica* genome (PRJNA230515 assembly in https://www.ncbi.nlm.nih.gov/

bioproject/PRJNA230515 ) using BLASTN (e-value 1e-4) and featured in Additional File 1: Table S1. The *F. gigantica* genome coordinates of previously published *F. hepatica* precursor miRNAs recently determined by Fontenla et al [43] are included in Additional File 1: Table S1.

### Expression Analysis

Differential expression analysis of the miRNAs was performed using normalised reads (counts per million; CPM). Hierarchical clustering of the total miRNAs and sample types was performed using One Pearson correlation using the average expression of the miRNA within the sample types, which was graphically represented using heatmaps generated using R (Morpheus package). Principal components analysis (PCA) of the normalised reads (CPM) was performed to determine clustering between miRNA expression of the sample types where the node size represents relative comparison of the number of miRNAs associated to each life cycle stage. Three-dimensional PCA plots were generated using the Partek suite for data visualisation. Expression of the miRNAs categorised based on miRBase, published and newly discovered were compared using parallel coordinates generated using Plotly. Expression of the top 10 most abundant miRNAs (CPM) was compared between each life cycle stage and pie charts generated using R (ggplot2 package).

### Target Prediction and Transcriptome Correlation

The target prediction tools miRanda and TargetScan were selected based on the consideration that they outperform other tools when used in combination and have shown the largest effect in supporting validation of targets [83]. Mature *F. hepatica* miRNAs were aligned to 3’UTR sequences derived from the *F. hepatica* genome (PRJEB25283) using the bioinformatic tool miRanda (v3.3a) where miRNA:3’UTR interactions with <-20 Energy-Kcal/Mol and >155 prediction score was accepted as authentic. These parameters were chosen as they are reportedly more stringent than default parameters for miRanda and have been previously used to help eliminate false positives for target prediction [84]. Additionally, 3’UTR sequences were aligned to all mature miRNA seed sequences using TargetScan (v7) where miRNA:3’UTR binding interactions of 7mer-8m site types were accepted. Gene expression analysis was performed for the gene targets commonly predicted by both tools based on the transcriptome data of the NEJ 24h, immature fluke 21 dpi and adult parasites generated by [35]. The gene transcripts corresponding to the predicted 3’UTR sequence targets were mapped to the transcriptome data using Salmon (v.13) and read counts extracted using htseq-count. Normalised gene expression (TPM) in each life stage was then correlated to the expression of their respective miRNA interactions (CPM) in each life stage using One Pearson correlation. Gene targets that had a negative correlation to miRNA expression (<0 correlation coefficient) with P value <0.05 were selected as the final predicted gene targets.

To correlate the data with our previous *F. hepatica* transcriptome and proteome studies of the NEJ, immature and adult parasite stages, the gene models derived from the newly revised *F. hepatica* genome (PRJEB25283) were mapped to the original draft *F. hepatica* genome (PRJEB6687). The genes predicted to be targeted by the miRNAs were annotated based on the functional annotation of the gene models by Cwiklinski et al. [35]. Hypergeometric tests were used to test for over-representation of GO terms within groups of genes associated with miRNA expression using R. Analysis of the genes involved in metabolism was based on our previous studies of the life cycle stage specific transcriptomes [30, 32, 35].

### Data Availability

The miR-Seq read data is freely available and deposited in NCBI’s GeneExpression Omnibus; accession number GSE186948. The *F. hepatica* genome and transcriptome data is freely available from WormBase ParaSite and the European Nucleotide Archive under accessions LN627018-LN647175 (assembly data), PRJEB6687/PRJEB25283 (genomic read data) and PRJEB6904 (transcriptomic read data).

## Supplementary Information

**Additional file 1: Table S1.** Sequences of Fasciola hepatica miRNAs and their genomic coordinates and features.

**Additional file 2: Table S2.** Revised annotation of Fasciola hepatica miRNAs across literature.

**Additional file 3: Table S3.** Raw and normalised (cpm) read counts for Fasciola hepatica miRNAs within newly excysted juveniles (NEJ 24h), immature (Juv) and adult (Ad).

**Additional file 4: Table S4.** Predicted targets of  Fasciola hepatica miRNAs using bioinformatic tool miRanda.

**Additional file 5: Table S5.** Predicted targets of Fasciola hepatica miRNAs using bioinformatic tool TargetScan.

**Additional file 6: Table S6.** All unique miRNA:mRNA interactions commonly determined by miRanda and TargetScan.

**Additional file 7: Table S7.** Summary of Fasciola hepatica miRNAs and their negatively correlated predicted targets, including functional characterisation. 

**Additional file 8: Table S8.** Gene ontology enrichment of negatively correlated gene target clusters.

**Additional file 9: Table S9.** Gene ontology enrichment of gene targets associated to life stage specific Fasciola hepatica miRNAs

**Additional file 10: Table S10.** All negatively correlated predicted gene targets associated to life stage specific Fasciola hepatica miRNAs.

**Additional file 11: Table S11.** MicroRNAs associated with Fasciola hepatica metabolism

**Additional file 12: Table S12.** miRNAs targeting genes within the aerobic metabolic pathways, graphically represented in Fig. 8.

**Additional file 13: Table S13.** Negatively correlated predicted targets of abundant Fasciola hepatica miRNAs.

**Additional file 14: Table S14.** Comparison of miRNAs and their predicted gene targets using miRanda and TargetScan.

**Additional file 15: Figure S1.**  Hairpin loop pre-miRNA structures using miRDeep2. Precursor miRNA structures determined from bioinformatic tool mirdeep2 (v2) and selected based on the presence of the predicted mature miRNA (red) in all triplicates of a life stage and the genomic coordinates of the precursor structure consistent across all the samples. Mature miRNA sequence is separated from complimentary star strand (blue) by sequences that make up the hairpin loop (orange). Nucleotides within the predicted star strand that were observed in sequencing data (purple) are also highlighted. Calculated minimum free energy (mfe) generated using RNAfold.

**Additional file 16: Figure S2.** Precursor miRNA sequences of Fasciola hepatica (fhe) (red), Schistosoma japonicum (sja) S. mansoni (sma) were compared using t-coffee multiple alignment tool and phylogenetic chart constructed using itol v6 (itol.embl.de).

**Additional file 17: Figure S3.** Frequency distribution histogram of miRNAs and their total number of predicted targets determined by both miRanda and TargetScan. Target number in bins of 20.

**Additional file 18: Figure S4.** Target prediction and analysis pipeline. Gene targets of Fasciola hepatica miRNAs are predicted using bioinformatic tools miRanda v2 and TargetScan v7. Common targets determined by predictive tools are then quantified in the transcriptome of each life stage using in silico data featured in Cwiklinski et al. [29]. Targets that are negatively correlated to their respective miRNA interaction are then used for analysis of all global miRNA -mRNA interactome, life stage specific miRNA,s top 10 abundant miRNAs and top 20 frequently targeted genes.

**Additional file 19: Figure S5.** bBoinformatic pipeline for detection and quantification of Fasciola hepatica miRNAs. Total RNA from intra-mammalian life stages newly excysted juveniles (NEJ) 24 hours post excystment, immature 21 days post infection (pi) and adults 16 weeks pi were extracted for library preparation and sequencing (*n* = 3). Reads were trimmed of adapters and filtered for low quality sequences and reads <18 nt long. Subsequent sequences were quantified for mature miRNAs featured in mirbase.org (version 22) and other published miRNAs featured in Ricafrente et al. [35]. Cleaned reads were also analysed for novel miRNAs structures and quantified using the mirdeep2 with inputs including the F. hepatica genome (accession: prjeb25283) and mature miRNAs of F. hepatica (miRBase and published), Caenorhabditis elegans, Schistosoma japonicum and Schistosoma mansoni (mirbase.org version 22).

## Data Availability

All data generated of analysed during this study are included in this published article [and its supplementary information files]

## Abbreviations

HSP: Heat shock protein
miRNA: Micro-RNA
NEJ: Newly excysted juvenile
SACMAD-1: Spindle assembly checkpoint component MAD1
