# Supplementary figures and images for: Stage-specific miRNAs regulate gene expression associated with growth, development and parasite-host interaction during the intra-mammalian migration of the zoonotic helminth parasite Fasciola hepatica
Source: BMC Genomics. 2022 Jun 4;23:419. doi: 10.1186/s12864-022-08644-z (PMC9167548; doi:10.1186/s12864-022-08644-z)

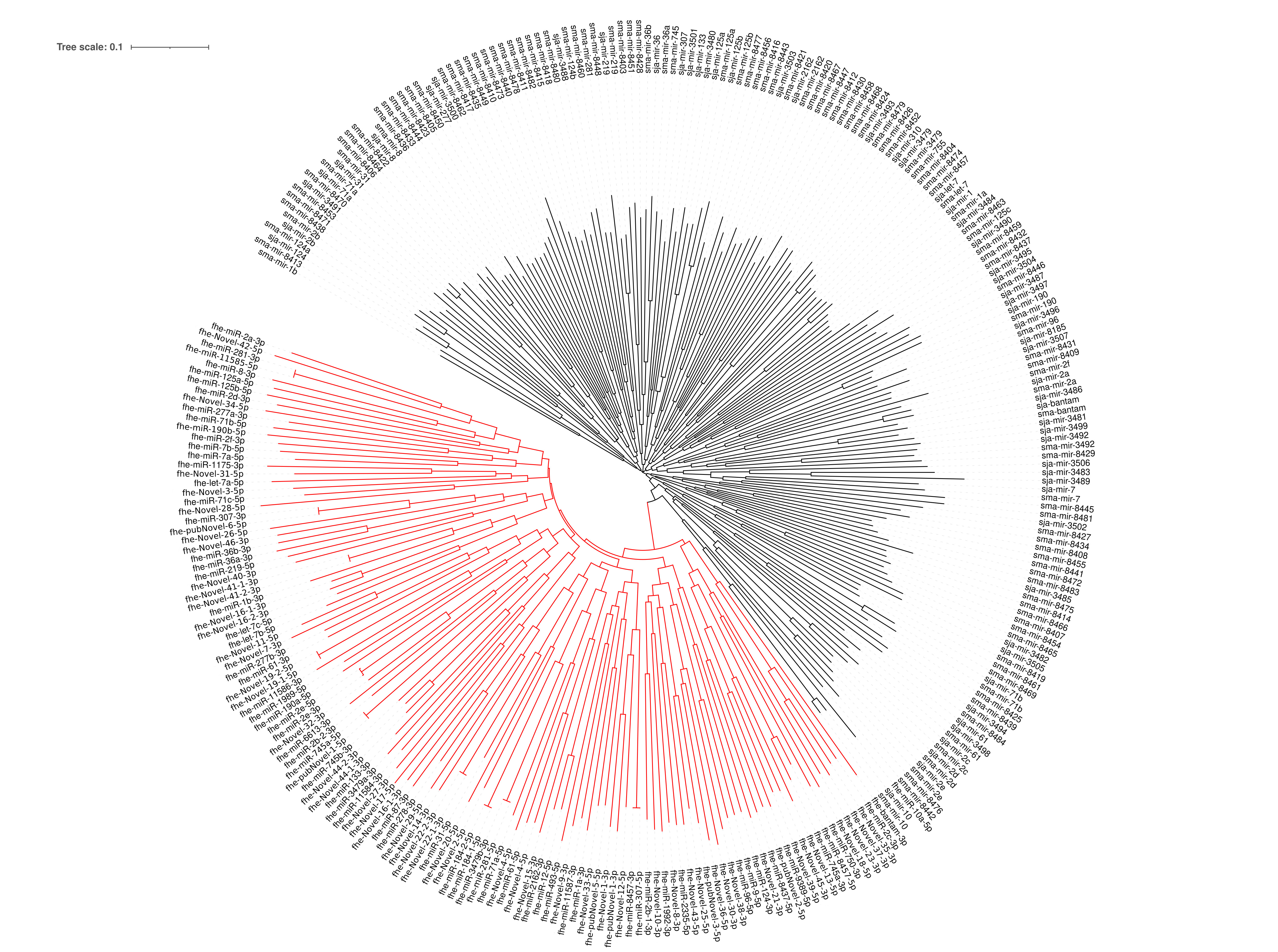

Supplement: Supplementary file 16 — Additional file 16: Figure S2. Precursor miRNA sequences of Fasciola hepatica (fhe) (red), Schistosoma japonicum (sja) S. mansoni (sma) were compared using t-coffee multiple alignment tool and phylogenetic chart constructed using itol v6 (itol.embl.de). [file 12864_2022_8644_MOESM16_ESM.jpg]

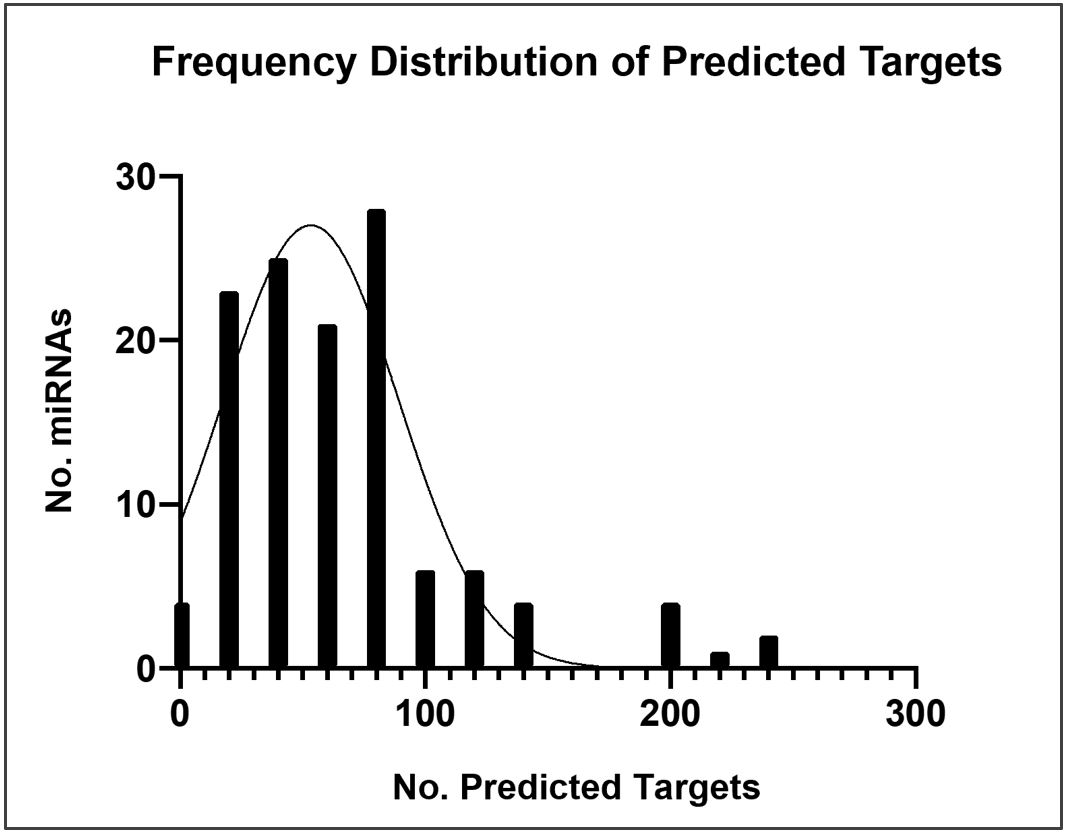

Supplement: Supplementary file 17 — Additional file 17: Figure S3. Frequency distribution histogram of miRNAs and their total number of predicted targets determined by both miRanda and TargetScan. Target number in bins of 20. [file 12864_2022_8644_MOESM17_ESM.jpg]

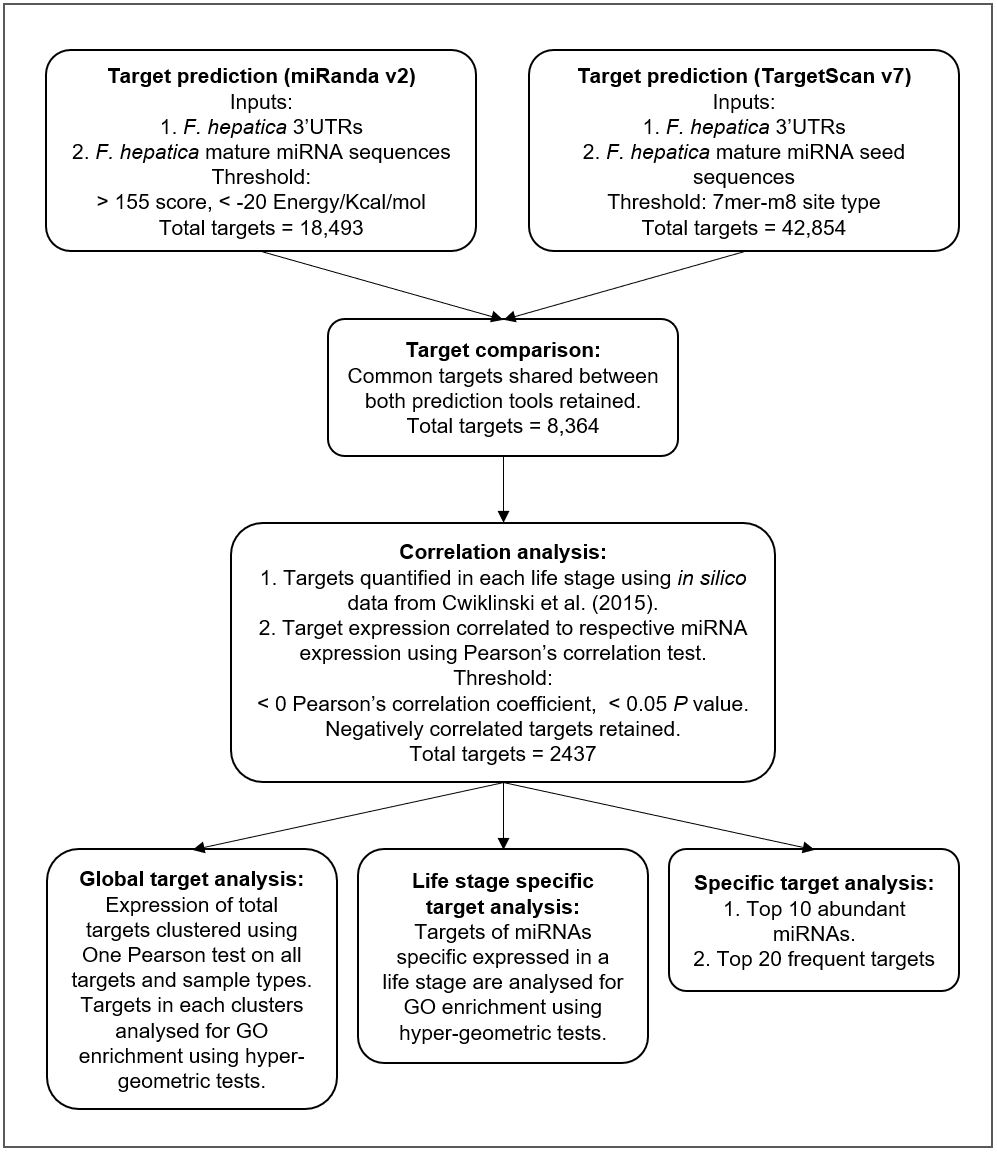

Supplement: Supplementary file 18 — Additional file 18: Figure S4. Target prediction and analysis pipeline. Gene targets of Fasciola hepatica miRNAs are predicted using bioinformatic tools miRanda v2 and TargetScan v7. Common targets determined by predictive tools are then quantified in the transcriptome of each life stage using in silico data featured in Cwiklinski et al. [29]. Targets that are negatively correlated to their respective miRNA interaction are then used for analysis of all global miRNA -mRNA interactome, life stage specific miRNA,s top 10 abundant miRNAs and top 20 frequently targeted genes. [file 12864_2022_8644_MOESM18_ESM.jpg]

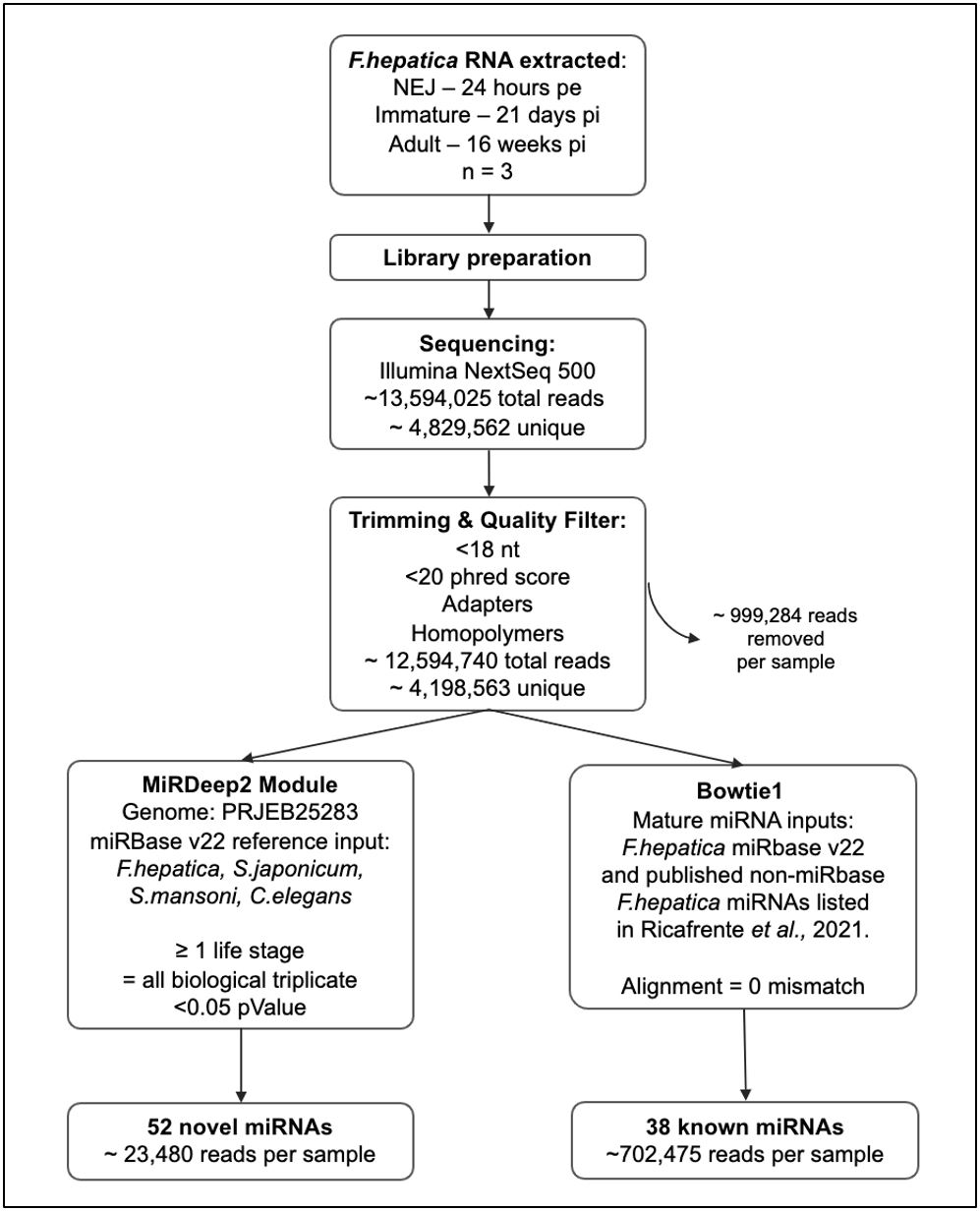

Supplement: Supplementary file 19 — Additional file 19: Figure S5. bBoinformatic pipeline for detection and quantification of Fasciola hepatica miRNAs. Total RNA from intra-mammalian life stages newly excysted juveniles (NEJ) 24 hours post excystment, immature 21 days post infection (pi) and adults 16 weeks pi were extracted for library preparation and sequencing (n = 3). Reads were trimmed of adapters and filtered for low quality sequences and reads <18 nt long. Subsequent sequences were quantified for mature miRNAs featured in mirbase.org (version 22) and other published miRNAs featured in Ricafrente et al. [35]. Cleaned reads were also analysed for novel miRNAs structures and quantified using the mirdeep2 with inputs including the F. hepatica genome (accession: prjeb25283) and mature miRNAs of F. hepatica (miRBase and published), Caenorhabditis elegans, Schistosoma japonicum and Schistosoma mansoni (mirbase.org version 22). [file 12864_2022_8644_MOESM19_ESM.jpg]
